# Uncertainty, Anxiety and Isolation: Experiencing the COVID-19 Pandemic and Lockdown as a Woman with Polycystic Ovary Syndrome (PCOS)

**DOI:** 10.3390/jpm11100952

**Published:** 2021-09-25

**Authors:** Lou Atkinson, Chris Kite, Gordon McGregor, Tamsin James, Cain C. T. Clark, Harpal S. Randeva, Ioannis Kyrou

**Affiliations:** 1 School of Psychology, College of Health and Life Sciences, Aston University, Birmingham B4 7ET, UK; jamestno@aston.ac.uk; 2 Warwickshire Institute for the Study of Diabetes, Endocrinology and Metabolism (WISDEM), University Hospitals Coventry and Warwickshire NHS Trust, Coventry CV2 2DX, UK; c.kite@chester.ac.uk (C.K.); ad0183@coventry.ac.uk (C.C.T.C.); 3 Centre for Active Living, University Centre Shrewsbury, University of Chester, Shrewsbury SY3 8HQ, UK; 4 Centre for Sport, Exercise and Life Sciences, Research Institute for Health & Wellbeing, Coventry University, Coventry CV1 5FB, UK; ac4378@coventry.ac.uk; 5 Centre for Exercise & Health, Department of Cardiopulmonary Rehabilitation, University Hospitals Coventry and Warwickshire NHS Trust, Coventry CV2 2DX, UK; 6 Warwick Clinical Trials Unit, Warwick Medical School, University of Warwick, Coventry CV4 7AL, UK; 7 Centre for Intelligent Healthcare, Coventry University, Coventry CV1 5FB, UK; 8 Warwick Medical School, University of Warwick, Coventry CV4 7AL, UK; 9 Aston Medical School, College of Health and Life Sciences, Aston University, Birmingham B4 7ET, UK

**Keywords:** polycystic ovary syndrome, PCOS, COVID-19, lockdown, lived experience, mental health, qualitative study

## Abstract

**Background:** The COVID-19 pandemic and the related lockdown measures presented a significant risk to physical and mental wellbeing in affected populations. Women with polycystic ovary syndrome (PCOS) are predisposed to several cardio-metabolic risk factors which increase the susceptibility to severe COVID-19 and also exhibit increased likelihood of impaired mental health wellbeing. Therefore, these women who usually receive care from multiple primary and specialist healthcare services may be disproportionately impacted by this pandemic and the related restrictions. This study aimed to explore the lived experience of the first UK national lockdown as a woman with PCOS. **Methods:** As part of a larger cross-sectional study, 12 women with PCOS living in the UK during the first national COVID-19 lockdown were recruited to a qualitative study. Telephone interviews were conducted in June/July of 2020, and data collected were subjected to thematic analysis. **Results:** Five themes were identified. “My PCOS Journey” describes participants’ experiences of diagnosis, treatment and ongoing management of their PCOS. “Living Through Lockdown” describes the overall experience and impact of the lockdown on all aspects of participants’ lives. “Self-care and Managing Symptoms” describe multiple challenges to living well with PCOS during the lockdown, including lack of access to supplies and services, and disruption to weight management. “Healthcare on Hold” describes the uncertainty and anxiety associated with delays in accessing specialised healthcare for a range of PCOS aspects, including fertility treatment. “Exacerbating Existing Issues” captures the worsening of pre-existing mental health issues, and an increase in health anxiety and feelings of isolation. **Conclusion:** For the women with PCOS in this study, the COVID-19 pandemic and the first national lockdown was mostly experienced as adding to the pre-existing challenges of living with their condition. The mental health impact experienced by the study participants was increased due to lack of access to their normal support strategies, limitations on healthcare services and uncertainty about their risk of COVID-19.

## 1. Introduction

In March 2020, the World Health Organization (WHO) declared a pandemic outbreak of the Coronavirus Disease-2019 (COVID-19) [1,2], caused by the severe acute respiratory syndrome coronavirus-2 (SARS-CoV-2) [3,4]. Most COVID-19 cases remain asymptomatic or mild, but severe symptoms and complications may develop in a proportion of predisposed/high-risk patients [3,4]. Due to this risk of severe and even fatal COVID-19, governments and relevant public health authorities worldwide enforced nationwide lockdown/quarantine measures, aiming to decrease the transmission of SARS-CoV-2 among the general population, and hence protect the health of vulnerable individuals [4,5,6,7]. Based on robust clinical and epidemiological evidence, it is now clear that, in addition to older age and male sex, susceptibility to severe COVID-19 is directly associated with a number of chronic cardio-metabolic diseases, such as obesity, diabetes, hypertension and metabolic syndrome [8,9,10,11,12,13,14]. Of note, with the exception of advanced age and male sex, the same cardio-metabolic risk factors which predispose to severe COVID-19 are also highly prevalent among women with polycystic ovary syndrome (PCOS) [15,16], placing this female patient population at potentially increased risk during the COVID-19 pandemic [17].

PCOS represents the most frequent endocrine disorder among women of reproductive age, with reported prevalence rates of 10–15% or even higher, depending on the utilised diagnostic criteria and the studied population/country [15,16]. Prominent features/symptoms of PCOS include ovarian dysfunction with chronic oligo-/anovulation and hyperandrogenism (either clinical with hirsutism, acne and androgenic alopecia, and/or biochemical with high circulating testosterone/androgen levels), whilst polycystic ovary morphology on ultrasound scanning may be also present [15,16,18]. As aforementioned in the majority of women with PCOS, obesity and a constellation of obesity-related comorbidities (e.g., type 2 diabetes (T2DM) and obstructive sleep apnoea (OSA)) compliment the common presenting PCOS phenotypes [15,16,19,20]. Of further importance, PCOS management in routine clinical practice is typically fragmented between multiple healthcare services and specialities, including general practitioners (GPs), gynaecologists, infertility specialists, endocrinologists and dermatologists [16,21], whilst it is also characterised by marked gaps regarding prompt diagnosis and appropriate management/treatment [22,23].

To date, despite the high prevalence of PCOS and the potentially high risk of these patients for severe COVID-19 [17,24,25], there is a paucity of research on the potential effects of the COVID-19 pandemic and the related lockdown/quarantine measures on this female population. However, previous published qualitative research which explored the lived experience of PCOS has highlighted some aspects that are likely to be relevant to living with this chronic health condition through the circumstances posed by the COVID-19 pandemic. These include feelings of difference and isolation from “normal” women [26,27]; perceptions of their condition not being adequately addressed by their GPs/doctors; difficulties accessing specialist healthcare services, including fertility and mental health services [28]; and the importance of emotional support from both family/friends and healthcare professionals in coping with the psychological distress caused by the condition [29]. Additionally, qualitative research consistently documents unremitting and draining struggles to control body weight [28], frustration and difficulties in getting answers to questions about their condition and symptoms [30], and negative impacts on their work, social interactions and romantic relationships [29]. As such, the objective of the present study was to capture the unique patient experiences of women with PCOS during the pandemic lockdown, by obtaining novel qualitative data addressing the research question of; “What is the experience of living through the COVID-19 pandemic lockdown restrictions in the United Kingdom (UK), as a woman with PCOS?”, in order to better inform the clinical practice in primary and specialist care regarding the respective management needs and issues identified as important by these patients during this disrupting period.

## 2. Materials and Methods

### 2.1. Study Design

This qualitative interview study was nested within a cross-sectional online survey/study, which aimed to examine a range of physical and psychological effects of the COVID-19 pandemic on women with PCOS, as previously described [31]. The aim of including a qualitative element to this study was to elucidate the quantitative findings, and to provide further insight into the experience of living with PCOS during this pandemic and its associated restrictions. As such, a target to recruit 12 women was set for the present qualitative interview study as a sufficient sample size to provide data on a range of experiences, while minimising participant burden. Ethical approval was granted by the ethics committee of Coventry University (ref. number: P106195) and all participants provided consent for their participation in this study.

### 2.2. Study Participants

In total, 12 women were interviewed for the present study. All women reported having received a diagnosis of PCOS. The ages of the study participants ranged from 24 to 42 years. Two participants identified as Asian, and all others as White. The reported body mass index (BMI) ranged from 22.6 to 53.6 kg/m^2^, with ten participants reporting a current BMI over 30 kg/m^2^ (coexisting obesity). Further details about the participants are presented in Supplementary Materials Table S1.

### 2.3. Study Procedure

Participants were recruited via the online study survey. Following informed consent, information regarding the present interview study was provided after completion of the survey questions, using the survey software, Qualtrics^©^ XM (Qualtrics XM, Provo, UT, USA). Participants indicated specific consent to be interviewed via an additional form within the survey, presented after the relevant participant information. Details of all survey participants who indicated willingness to take part in an interview were passed to the lead author. In order to maintain anonymity, a mobile phone number was the only personal information collected from the survey participants, and subsequently participants were invited for an interview via SMS text messaging. The list of consented participants was worked through in date order until 12 participants had been recruited and interviewed.

### 2.4. Data Collection

Data collection took place between 8 June and 1 July 2020. Interviews were conducted by phone at a time agreed with the participant via text messaging. Telephone interviews are well suited to qualitative studies such as the present study, where the primary aim is to gather more detailed data pertaining to topics explored quantitatively [32] and have been shown to be an effective method of data collection, with some advantages over face-to-face interviews [33]. Additionally, face-to-face interviews would have been neither safe nor legal due to the stage of the pandemic response in the UK at that time. Telephone interviews also enabled participants to maintain their anonymity, which may not have been assured if video conferencing had been used. Interviews were audio recorded following consent by the participant and were then transcribed verbatim. All interviews were conducted by the lead author, a very experienced qualitative researcher specialising in women’s health. A topic guide was devised to explore the same topics as the quantitative questions, and this was used to guide each interview, although participants were encouraged to speak freely about anything they felt was relevant to their experiences. Duration of interviews ranged from 16 to 38 min, with a mean duration of 24 min.

### 2.5. Data Analysis

Data were imported into NVivo© (QSR International Pty Ltd., Chadstone, VIC, Australia) and analysed by the lead author using a process of inductive thematic analysis, following the principles outlined by Braun and Clarke [34]. In brief, the process involved initial familiarisation, via multiple readings of the transcripts, where initial thoughts about patterns within the data were noted. This was followed by line-by-line examination of each transcript, where all elements relevant to the research questions were identified and assigned a code, in order for extracts relating to the same subject to be collated together. Codes were then grouped together into initial themes, which were then checked against the dataset and refined, to produce a thematic map. Finally, themes were named and described, and data extracts were selected to illustrate the finalised themes. A realist approach was taken to data analysis, where data were treated as reflecting the participants’ reality, a priori knowledge and assumptions were set aside, and data were coded according to the prevalent ideas and content within.

## 3. Results

**Figure 1:**
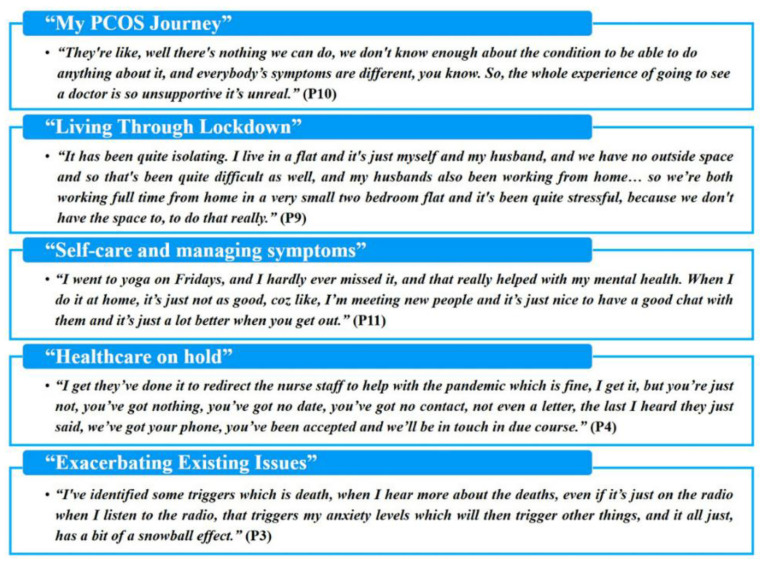
List of the five main themes that were derived from the study dataset with illustrative quotes (P: participant).

Five main themes were derived from the study dataset. These are briefly summarised in Figure 1. One theme, “Living Through Lockdown”, describes the experience of the pandemic and lockdown that were not specific to having PCOS. Another theme, “My PCOS Journey” describes participants’ experience of their condition prior to the pandemic, including symptoms, diagnosis, treatment and the social and emotional impact of the condition. While these themes offer important context, they will only be described briefly in order to focus on the remaining themes which describe the more unique experiences of living through the COVID-19 pandemic and lockdown as a woman with PCOS. These are: “Self-care and Managing Symptoms”, “Healthcare on Hold”, and “Exacerbating Existing Issues”.

### 3.1. My PCOS Journey

Every participant’s experience of PCOS was unique, but between the 12 interviewed participants the broad spectrum of PCOS symptoms was described, including absent, irregular and/or heavy and painful menstrual periods, difficulties with increased body weight and weight management, acne, excess hair growth, low energy, uncontrollable emotions, and low mood or depression. Of note, while some women reported being *“so lucky”* (P5) to have had no problems conceiving, others had been unsuccessful when trying to conceive naturally, and the majority reported worrying about their chances of having children, either presently or prior to conceiving.

*“when I found out that I only ovulated from one ovary I sat and cried…I was terrified.”*

(P8)

Moreover, a range of PCOS diagnosis experiences were reported. Some women experienced symptoms for years before being diagnosed, which was a source of frustration, as they commonly felt that they knew something was “wrong”. In contrast, among the participants who were diagnosed as a teenager or very young woman, several reported feeling disappointed with their healthcare experience following diagnosis. This included simply being prescribed the contraceptive pill, told not to worry, and/or being told they might struggle to conceive and to come back when they were ready to have children.

Descriptions of struggles to get support and treatment from healthcare professionals were common among participants, as were reports of having to push for and/or try different medications before finding an effective treatment for specific symptoms.

*“No one’s offered me an endocrinologist or a dermatologist I’ve just been told it’s not bad enough. And I was like well who are you to say it’s not bad enough? It’s bad enough for me.”*

(P5)

To these experiences, women often described searching elsewhere for information and treatment options and trying their own strategies to manage their condition.

*“the last couple of years I’ve spoken to a lot of other women, become a lot more knowledgeable I suppose, self-educated about it, probably more so in the last year… I was also trying to change my diet a bit during that time as well, I looked into supplements and things like that so probably since about August/September I’d start making some gradual changes in that respect as well, just sort of educating myself a bit more in the absence of any doctors.”*

(P2)

One of the challenges most commonly reported was with trying to lose weight, with 11 of the 12 participants describing an ongoing battle with their body weight. Some participants had been successful at losing weight and reported improvement in symptoms and quality of life as a result. However, others felt that nothing they tried had worked or perceived that the effort and restrictions required to maintain weight loss were much higher than for women who do not have PCOS, and therefore much more difficult to sustain.

*“I’ve done bootcamps, I’ve done the meal replacement diets, with varying success. Ten years ago, I managed to lose 8 stone because I didn’t eat any food other than the meal replacement packs for about six months. And it was fine, and it worked brilliantly, it was fantastic and there was a lot of symptoms cleared up and it was fab. They do really work and I’m trying it again actually and I lost 5 pounds last week, but its balance, you know sometimes I just want to be normal.”*

(P9)

Women frequently identified their weight, acne and hair growth as causes of the far-reaching psychological and social aspects of having PCOS, including low self-esteem, low sex drive and challenges with socialising or relationships. This was in addition to experiencing low mood and heightened emotions. As a result, many participants described how the accumulation of symptoms of PCOS had a significant detrimental and enduring effect on their mental health.

*“if it’s not the acne it’s the scars…and if it’s not that, it’s my weight and if it’s not that it’s the hair. If it’s not that it’s my mood, do you know what I mean, it’s like this spider web of things and you can’t sort find your way out of it.”*

(P10)

### 3.2. Self-Care and Managing Symptoms

This theme describes the practical and emotional impact of lockdown restrictions in relation to participants’ ability to manage their PCOS symptoms and engage in self-care.

#### 3.2.1. Access to Supplies and Services

The closure of shops, travel restrictions and worry about exposure to the coronavirus made it difficult for some women to access key supplies and services. These included sanitary products, medication, healthy food, dietary supplements and hair removal services.

*“when you’ve got access to shops, and chemists if you’re relying on long term medication, or if you’re struggling with certain menstrual problems accessing things like sanitary pads and stronger painkillers that’s been quite difficult, that’s been quite a challenge… if Tesco’s don’t have them in stock or if you’re not in an area where the pharmacy is going to deliver to you, that kinda thing, that’s been quite difficult.”*

(P11)

*“to begin with, food was like, it was hard to get hold of, do you know what I mean? You just sort of, bought what you could to keep you going and that included a lot of things that were, processed do you know I mean, like sausage rolls and all the bits in the cupboard.”*

(P10)

Women also reported that the inability to access exercise facilities, such as gyms, pools and classes, along with decreased physical activity due to travel restrictions and changes to daily routines, had severely impacted their ability to manage their physical and mental symptoms.

*“I would say not being able to do as much exercise. So, for example I can’t go to the gym, so that affects my weight as well as my mood. Not being able to go out as much affects my mood.”*

(P7)

For many participants, there was significant concern that their weight loss/management journey would be disrupted as a result of these changes.

*“I just didn’t want to go backwards in my weight loss plans and worsen my PCOS symptoms.”*

(P1)

*“I have gained three quarters of a stone since lockdown started, which is incredibly frustrating.”*

(P5)

*“I would say, yeah it’s made me want to comfort eat more if I’m honest. It just makes me want to comfort eat more, I think with me anyway, growing up with PCOS I found it really difficult in terms of some of the symptoms, especially the excess hair and I feel like as a coping mechanism I would comfort eat. And I stopped doing that, but now it’s coming back again because of the pandemic, and the anxiety around how life is changing in all these different ways. So I feel like I’m going back to using unhealthy coping strategies again. Sometimes you just don’t feel like making an effort with your appearance, coz you’re not going out either.”*

(P7)

An inability to access social support was also highlighted as detrimental to managing the mental health effects of PCOS during lockdown.

*“And in terms of the, just like emotions being able to get out and have a breather. Being able to talk to my friends and my family about these issues I can’t really do that, like see them face to face I can’t do that, go out and do that.”*

(P7)

#### 3.2.2. Freedom from Usual Pressures

A number of study participants noted positive impacts of the disruption to their usual daily lives. These included being more able to rest and to manage symptoms when working from home, as well as establishing new exercise and eating routines.

*“at the start you had kinda less access to certain foods, a lot less access to shops so that’s helped a bit this managing my health and wellbeing a bit. You’ve got to make more kinda smarter choices about what you’re eating I suppose. Walking everywhere as well instead of being able to use public transport so easily, so that’s helped a bit.”*

(P11)

*“I had like a couple of days where I’ve been really stressed and I’ve had like a bit of bleeding, but I’m alright because you know I can change like every hour. But when I’m at work or whatever I can’t and I need to have days off work. But when I’m at home I’m okay, well I think I am!”*

(P6)

Some women noted that they had had more time to do research and to think about their condition and their management of it. This break from the usual daily routine had also facilitated a change in some women’s perspectives of their lives and conditions.

*“I know I have been looking at diets and things more and trying to consider, like after all of this has happened, and I’m in a better state of mind to think about how I’m gonna consume food more... And I don’t know that if before all of this, I would have really sat and thought about it. For so much time.”*

(P8)

*“it’s given me a drive to actually lose weight, which is always good. And maybe speak to people more when I’m struggling.”*

(P12)

Of note, some women reported a reduction in anxiety as a result of not socialising, mostly due to the perceived stigma and social pressure associated with overweight/obesity and not meeting societal “beauty standards”.

*“If anything, I think it’s been quite good in the sense that I’ve been a little bit less paranoid about having to do things like hair removal and that kind of stuff, because I’m not going out as much. So, it’s removed some of that social anxiety regarding it.”*

(P5)

### 3.3. Living through Lockdown

Most study participants had switched to working from home, as had some of their partners, whilst those with children were also home schooling. The majority of participants were living with their partner and any children they had, while a few had moved back to their family home or that of their partner during the lockdown. Associated with these working and living arrangements, participants reported a range of negative social and emotional impacts. These included feeling lonely, isolated from friends and family, and a sense of not being able to escape the small world to which they were restricted.

*“I’ve got no choice really but to stay indoors more, so I think that’s… it’s frustrating. I think that emotionally can take a toll on you, sometimes I feel like socialising with, say like family members like my mum or dad and I don’t get to see them which makes me feel a bit isolated. And its little things like going to the shops and things because you feel more anxious about the time going, I just feel very anxious. And I think that can, and when you feel anxious and you are changing the way you live it makes me feel a bit like low mood as well. So yeah I think that’s it.”*

(P7)

Participants also described increased stress levels, poorer sleep, and worry about the coronavirus, both personally and for loved ones, making statements such as: *“when there’s a lot of people dying and you hear about those people that have got the virus I do think, it is a scary situation”* (P3); and *“For myself I’m not worried about it, but for my family I am.”* (P9)

### 3.4. Healthcare on Hold

Participants described experiencing reduced access to healthcare professionals and services during the COVID-19-related lockdown. This included access to their usual clinicians, as well as delays to services they were waiting to receive pre-pandemic. Both of these experiences were sources of significant anxiety and distress for these women, delaying them from further understanding and overcoming/managing their symptoms.

*“I had a period this week and because it was quite heavy and painful, I had phoned the GP surgery and I’d said if I could be given something like tranexamic acid even, just to make things a bit more manageable, but to actually get that help, you have to fill out an online form for the surgery, who then discuss it with the doctor, and then the doctor tells the receptionist what to do, then the receptionist phones me back, but they said that process could take up to three to four weeks, so that, yeah you feel that you can’t get the advice you would normally get at all.”*

(P11)

*“I’m not able to be in touch with my doctor as much as I’d like, because she gives me some really good advice on things that she thinks will help, and obviously everybody is like drowning into the doctor’s system, so it’s really difficult to see anyone. And I absolutely adore my doctor she’s been amazing to me.”*

(P8)

Women who were waiting for fertility treatment found this particularly frustrating, as they had often been working towards getting that treatment for a long time.

*“We’re waiting on an appointment for [fertility] treatment …, just before it happened and now everything’s put on hold… When you ring up to find out anything it’s just automated message that says we’ll come back to you, and it’s really hard coz we’ve already waited two and a half years to get to this point in March, and it’s just been pulled from under our feet again.”*

(P4)

Additionally, there was concern from some women that if they needed healthcare in the future, including fertility treatment, this would be much more difficult to access.

*“I thought about things like, if in the future I needed IVF because maybe I might find it difficult to get pregnant, in the future maybe because of my PCOS because I know these things can change. How would that work out, in terms of me being able to do that? It made me think about accessing, for example accessing like GP support really around my PCOS, I can’t just go to the doctor and see someone face to face.”*

(P7)

### 3.5. Exacerbating Existing Issues

For many participants, the pandemic worsened their pre-existing mental health issues. Indeed, several participants described anxiety about the coronavirus, stress of a changed working environment, financial issues and being in constant close proximity to others in their household as exacerbating their mood swings, anxiety and depression.

*“at home it is not really somewhere I can relax and I’m very tense and very anxious and a lot of that is to do with my mood and my depression that I get with my PCOS symptoms. So obviously spending all of my time at home and not being able escape and to go the gym or several dog walks a day or whatever I was finding it really hard, and I was kind of going backwards in a lot of things.”*

(P1)

*“with me having so much time on my hands to think. There are times, you know over the last three months where I’ve had to sort of have a word with myself, do you know what I mean and sort of turn myself off and tell myself to calm down, sort of talk myself out of it, you know because that, that panic is just bubbling underneath, just there you know, and it’s like, the bars only gotta be shortened a little bit before it explodes, you know so it’s very hard.”*

(P10)

Additionally, a few participants mentioned that they felt more vulnerable to the coronavirus because of their PCOS or were uncertain as to whether they were at more risk.

*“I am really worried about catching it, like cos I haven’t seen a lot of things, you know like regarding PCOS and the virus so I would like to have seen a bit more things about it, because obviously there is quite a few women with it, they wouldn’t have seen as well which is really worrying like, am I gonna get it. I know you can’t tell if you’re going to get it really bad, but are we in that category where it could be really bad?”*

(P6)

Although one woman reflected that living with a chronic condition may have prepared her for the pandemic experience, she also felt vulnerable.

*“I think in that sense, the pandemic in some ways is easier to cope [with] because you’re sort of used to that level of restriction on your life anyway, but in other ways, I think it can be much more frightening as well because you realise that you’re probably one of those people who’s going to be a bit more vulnerable to that virus, and more vulnerable to not being able to access healthcare so easily as well, so I think it’s yes, there’s both kind of a positive side of having the sort of long term health issue, it can have a positive influence on how you cope with this kind of situation, but it has quite a negative one as well, it’s kind of pretty mixed.”*

(P11)

Whether due to a sense of increased vulnerability, or through more general caution about protecting themselves and their families from COVID-19, many participants described severely limiting their social contact during the lockdown. This had inevitably increased the sense of isolation and separation from the social world which many had reported when describing their pre-pandemic PCOS journey.

*“we may try and socially distantly see my parents in the next few weeks. But we’ve not seen them at all, they’re over in Yorkshire so we would have had to drive to see them so it wasn’t an option anyway until recently, and we’ve sort of held back on that a bit longer. But yeah certainly keeping in touch with everyone, I just think that “I know my friends aren’t seeing each other separately anyway” we’re all sort of spread out and things, so I don’t feel that I’m missing out on things that, that my friends are doing, same with my family. So yeah, it’s always been fine really yeah, I’ve had days where I’ve felt a little bit down. I suppose, you just get a bit cabin fever.”*

(P2)

*“I think I’ve been out four times since lockdown began, which has impacted my mental health quite a bit.”*

(P8)

## 4. Discussion

The women in this study described a “PCOS journey” which mirrored the lived experiences of PCOS documented in previous research. This includes frustration with primary care services including late diagnosis [29], difficulties accessing treatment and lack of support from healthcare professionals post-diagnosis [28]. As other research has also found, the study participants described significant and enduring difficulties in losing/managing body weight [28], as well as a wide range of mental health issues and detrimental impacts on relationships, work and social lives [29]. Most notably, in line with prior research findings, the participants in this study also described feeling isolated and different from other women [26,27] before being physically isolated by restrictions on social contact. The women in this study reported aspects of ”living through lockdown” that have also been reported within the general UK population’s experiences of the COVID-19 pandemic. These included loneliness [35], missing face-to-face contact with friends and family [36], disruption of normal routines [37], anxiety for their own and others’ health [37], and feelings of distress or depression as a result of feeling trapped or claustrophobic by the restrictions [36].

Uniquely, the participants in this study revealed the impact of the pandemic and lockdown restrictions on them as women with PCOS. This included changes to how they ”managed symptoms and self-care”, experienced delays and uncertainty due to ”healthcare on hold”, and particularly in relation to emotional wellbeing and mental health, an ”exacerbation of pre-existing issues”. Within these experiences, some potentially positive impacts can be identified; of note, a few women had felt a freedom from pressure to achieve a certain aesthetic, as they were not being seen by many people outside of their household. Previous studies have identified that women with PCOS who experience visible symptoms/signs, such as hirsutism, acne or obesity, report feeling that they “lack femininity” or “feel ugly” [26,27]. In turn, this can lead to low self-esteem and eating disorders [38] and can also be a significant barrier to engaging in physical activity in public settings [39]. However, this reduction in social anxiety is likely to provide only temporary relief until restrictions on social contact are removed and social interactions become more frequent. Homeworking was also identified as positive for some women in managing their PCOS, as it gave them flexibility to rest or take a break from work in response to their symptoms. Early indications are that many employees will be able to continue with some element of flexible working or homeworking once government guidance to work from home has been lifted [40], which may provide some women with PCOS a working environment that better enables them to manage their symptoms; although, downsides of homeworking (e.g., prolonged social isolation and an inability to detach from work) should be well-considered and mitigated in this context prior to decisions for future homeworking arrangements [41]. Additionally, some participants reported that the lockdown had afforded them time to research PCOS management, or an opportunity to reconsider their own approach to PCOS management, including making positive lifestyle changes. This could lead some women to emerge from this pandemic with an improved quality of life in relation to their PCOS. Nevertheless, this has once again highlighted that many women with PCOS feel that adequate primary healthcare services are lacking and that they are solely responsible for working out how best to manage and improve their condition.

Despite these few prospective positive outcomes, overall, the findings of the present study paint a picture of a very challenging experience for women with PCOS during the pandemic and lockdown restrictions. Immediate impacts included the distress experienced as a result of difficulties in readily purchasing sanitary products and pain medication as needed. The irregularity of menstruation that is a common symptom of PCOS increases the likelihood that these participants would require supplies with little or no warning, and this combined with the often heavy and painful periods experienced by women with PCOS resulted in much more than a minor inconvenience for these participants. Of further concern is the worsening of mental health reported by many participants. Participants described how the low mood or mood swings, anxiety and/or depression they experienced as part of their PCOS had become worse during the COVID-19 pandemic. Prior research has identified that the women with PCOS are more likely to experience depression, anxiety and perceived stress than women without PCOS [42], and that the majority of women with PCOS experience mental health symptoms [28]. Participants in this study attributed a worsening of these symptoms partly to reduced access to social support, and previous research has identified emotional support from friends, family and other women with PCOS as an important coping strategy [29]. However, some participants also highlighted feelings of vulnerability to the coronavirus, or uncertainty about their level of risk based on their condition. PCOS is not mentioned as a condition that may increase risk or require “shielding” by any of the UK government coronavirus guidance issued in 2020. Yet, conditions that are highly prevalent within the female population with PCOS, such as obesity and diabetes, were identified as increasing risk of severe illness and death from COVID-19 early in the pandemic [43]. Indeed, participants of this study mentioned their body weight, insulin resistance and/or increased risk of developing diabetes and questioned whether this meant that their PCOS places them at higher risk for COVID-19. This lack of adequate information and risk-assessment regarding the potential higher risk of COVID-19 in individuals with PCOS has been highlighted as a concern for clinical practice [17] and was identified as a cause of significant health anxiety for many participants in this study.

Substantial additional distress was caused by restricted access to primary care and a pause in certain other specialist healthcare services relevant to PCOS, and the uncertainty of when these would be restored. Previous studies have highlighted that women with PCOS report feeling they have to fight for specialist healthcare for their condition [44]. The impact of this reduced capacity for PCOS treatment/management on the women in the present study ranged from frustration and disappointment, to distress and anxiety, as often women felt they had been left waiting with no certainty of when such services would be available again. There were also specific worries that time may be running out for those awaiting fertility treatment. While this only applied to a couple of the 12 women in the present study, a recent UK survey also identified that cancellation of fertility treatment during the COVID-19 pandemic has caused significant psychological stress, feelings of isolation and weakened coping mechanisms in female fertility patients [45]. Some participants also mentioned that their healthcare professionals (e.g., primary care physicians) were an important source of support and that they were currently unable to easily contact them or have face-to-face appointments. Finally, participants highlighted the negative effect on their lifestyles due to the COVID-19 pandemic and lockdown restrictions, including poor sleep, less access to healthy food and dietary supplements, and closure of exercise and leisure services. In such a patient group with a chronic condition for whom weight management is both highly needed, as a means to improve symptoms and/or to increase the chances of conceiving a child, and very challenging due to the underlying PCOS pathophysiology (e.g., vicious cycle between hyperandrogenism, central obesity and insulin resistance) [15,16], the impact of these changes on participants’ wellbeing cannot be underestimated. Indeed, several participants expressed concerns about gaining weight or disruption to ongoing weight loss management. As such, it is evident based on all these findings that a multitude of factors have presented significant challenges to these participants’ mental wellbeing and have the potential for long-term deleterious consequences.

In comparison to evidence gathered at a similar time for the impact of the COVID-19 pandemic and lockdown restrictions on other populations with metabolic conditions, a qualitative study of adults with diabetes in Denmark reported similar challenges to self-management of the condition, including access to exercise and emotional eating [46]. The findings of this study also identified anxiety relating to fear of the coronavirus and concerns about access to medication, and some participants reported that the lockdown gave them time to learn more about their condition. However, in contrast to the present study, the authors reported that some participants had experienced little change to their daily lives and were relatively unconcerned about the impact on their wellbeing. In another qualitative study in Ireland involving participants undergoing treatment for obesity, similar disruption to maintaining physical activity and dietary behaviours were reported, with a similar smaller number of positive changes to diet also noted [47]. In this study, the psychological impact was more evident and similar to the present study, with participants reporting anxiety and fear, both of the coronavirus and in relation to their weight loss goals [47]. Perhaps the most similar finding however, can be found in studies of individuals with pre-existing mental health conditions, also conducted in the summer of 2020. For example, a UK qualitative interview study of 49 individuals with a range of mental illnesses [48] noted that some participants experienced a relief from external pressures during lockdown, but also reported a worsening of their mental health due to reduced access to mental healthcare services, and disruption to their normal life and coping strategies, including social support and exercise. A pre-print report of another UK study [49] also identified some emotional relief from reduced social contact. However, these authors also report that isolation from social support, anxiety about the coronavirus and perceived lack of mental healthcare services, including worry about whether healthcare would be available in the future, all contributed to a deterioration in mental wellbeing in participants with pre-existing mental illness.

Certain limitations of the present study must be acknowledged. The participants in this study had agreed to participate in both the “parent” cross-sectional research study and the present qualitative study, which were advertised via social media, including those of a PCOS charity (Verity, the UK PCOS charity) that offers online support forums and information for women with PCOS. As such, the study participants can be considered as motivated to contribute to research and may be more knowledgeable and/or engaged with their PCOS symptoms and treatment than other women with PCOS who do not seek out such information and support. However, this is common in such research studies, whilst the timely nature of the present research necessitated an expeditious recruitment method. Additionally, the large numbers of women who volunteered to participate in both studies suggests that the women with PCOS who are willing to contribute to research are not limited to only a highly motivated few. Moreover, as data were collected within a short timeframe during the first UK COVID-19 related lockdown, findings represent a snapshot of these women’s experiences at that time. Nevertheless, to our knowledge, this is the only study to explore the experience of the COVID-19 pandemic and lockdown restrictions for women with PCOS, and as such provides valuable insight which can be used to better support women with PCOS throughout this pandemic and beyond.

## 5. Conclusions and Implications for Practice

To our knowledge, this is the first qualitative study which captured the lived experience of women with PCOS during the COVID-19-related lockdown. The findings of the present study, categorised into five main themes as summarised in Figure 1, add to the growing body of evidence which highlights the struggles faced by women with PCOS, both in the UK and elsewhere (e.g., problems in accessing the primary and specialised healthcare services needed to treat the wide range of symptoms associated with this chronic condition). Indeed, the additional delays and uncertainty caused by the COVID-19 pandemic has caused significant distress to this frequently overlooked female patient population. In the UK, GPs are both the first port of call for health queries and gatekeepers to specialised healthcare, and as such these first-line primary healthcare practitioners should aim to encourage women with PCOS to continue to seek help when needed. Both GPs and specialist healthcare providers should make targeted efforts to keep such vulnerable patients informed about timescales for required treatments and specialist appointments. This further includes services such as fertility treatment, which may be both limited in availability for many and time sensitive. In the longer term, it appears clear that women with PCOS feel poorly understood and underserved by the current healthcare provision, as has been further highlighted during the COVID-19 pandemic. Given its prevalence, a fresh approach where women with PCOS are treated holistically and not as a disparate set of symptoms, along with better education of health professionals about the condition, would improve health outcomes and the quality of life for many women with PCOS, both under routine conditions and under emergency situations such as those posed by the COVID-19 pandemic.

Weight management is a pervasive challenge for the participants in this study, and the COVID-19 pandemic risked undoing the progress they had made. As indicated by the participants who found this pandemic to be an opportunity to research, re-evaluate and change their lifestyle, moving forward it is likely that behaviour change interventions would be extremely valuable to enable women with PCOS to make and sustain lifestyle change. This can be supported at scale by weight management services within primary care.

The participants in this study demonstrated a worsening of their mental health overall. Participants were uncertain of how PCOS affected their COVID-19 risk, and this added to the anxiety and low mood or depression which was exacerbated by social isolation and the inability to employ their usual coping strategies, such as social support and physical activity. Given the high prevalence of depression, anxiety and other mental health disorders in women with PCOS, this is a patient group who would benefit from urgent and continued support from mental healthcare services [50], as well as clearer communication from primary care physicians regarding their ongoing risk as we move towards easing restrictions and “living with the coronavirus”.

## Data Availability

Further details are available via the corresponding authors upon reasonable request, and where it is ethically acceptable to do so, and does not violate the protection of study participants, or other valid ethical, privacy, or security concerns.

## Supplementary Materials

The following are available online at https://www.mdpi.com/article/10.3390/jpm11100952/s1, Table S1: Participant characteristics collected via online survey.

## References

[1] Cucinotta D., Vanelli M. (2020). WHO Declares COVID-19 a Pandemic. Acta Biomed..

[2] WHO (2020). Coronavirus Disease 2019 (COVID-19): Situation Report, 51. https://www.who.int/emergencies/diseases/novel-coronavirus-2019/situation-reports.

[3] Bohn M.K., Hall A., Sepiashvili L., Jung B., Steele S., Adeli K. (2020). Pathophysiology of COVID-19: Mechanisms Underlying Disease Severity and Progression. Physiology.

[4] Osuchowski M.F., Winkler S.M., Skirecki T., Cajander S., Shankar-Hari M., Lachmann G., Monneret G., Venet F., Bauer M., Brunkhorst M.F. (2021). The COVID-19 puzzle: Deciphering pathophysiology and phenotypes of a new disease entity. Lancet Respir. Med..

[5] Nussbaumer-Streit B., Mayr V., Dobrescu A.I., Chapman A., Persad E., Klerings I., Wagner G., Sieber U., Christof C., Zachariah C. (2020). Quarantine alone or in combination with other public health measures to control COVID-19: A rapid review. Cochrane Database Syst. Rev..

[6] Girum T., Lentiro K., Geremew M., Migora B., Shewamare S. (2020). Global strategies and effectiveness for COVID-19 prevention through contact tracing, screening, quarantine, and isolation: A systematic review. Trop. Med. Health.

[7] Caristia S., Ferranti M., Skrami E., Raffetti E., Pierannunzio D., Palladino R., Ancona C. (2020). Effect of national and local lockdowns on the control of COVID-19 pandemic: A rapid review. Epidemiol. Prev..

[8] Du P., Li D., Wang A., Shen S., Ma Z., Li X. (2021). A Systematic Review and Meta-Analysis of Risk Factors Associated with Severity and Death in COVID-19 Patients. Can. J. Infect. Dis. Med. Microbiol..

[9] Wingert A., Wingert A., Pillay J., Gates M., Guitard S., Rahman S., Beck A., Hartling L. (2021). Risk factors for severity of COVID-19: A rapid review to inform vaccine prioritisation in Canada. BMJ Open.

[10] Hamer M., Gale C.R., Kivimäki M., Batty G.D. (2020). Overweight, obesity, and risk of hospitalization for COVID-19: A community-based cohort study of adults in the United Kingdom. Proc. Natl. Acad. Sci. USA.

[11] Booth A., Reed A.B., Ponzo S., Yassaee A., Aral M., Plans D., Mohan D. (2021). Population risk factors for severe disease and mortality in COVID-19: A global systematic review and meta-analysis. PLoS ONE.

[12] Schlesinger S., Neuenschwander M., Lang A., Pafili K., Kuss O., Herder C., Roden M. (2021). Risk phenotypes of diabetes and association with COVID-19 severity and death: A living systematic review and meta-analysis. Diabetologia.

[13] Du Y., Zhou N., Zha W., Lv Y. (2021). Hypertension is a clinically important risk factor for critical illness and mortality in COVID-19: A meta-analysis. Nutr. Metab. Cardiovasc. Dis..

[14] Shah H., Khan M.S.H., Dhurandhar N.V., Hegde V. (2021). The triumvirate: Why hypertension, obesity, and diabetes are risk factors for adverse effects in patients with COVID-19. Acta Diabetol..

[15] Kyritsi E.M., Dimitriadis G.K., Kyrou I., Kaltsas G., Randeva H.S. (2017). PCOS remains a diagnosis of exclusion: A concise review of key endocrinopathies to exclude. Clin. Endocrinol..

[16] Kyrou I., Weickert M.O., Randeva H.S., Ajjan R., Orme S.M. (2015). Diagnosis and Management of Polycystic Ovary Syndrome (PCOS). Endocrinology and Diabetes: Case Studies, Questions and Commentaries.

[17] Kyrou I., Karteris E., Robbins T., Chatha K., Drenos F., Randeva H.S. (2020). Polycystic ovary syndrome (PCOS) and COVID-19: An overlooked female patient population at potentially higher risk during the COVID-19 pandemic. BMC Med..

[18] The Rotterdam ESHRE, ASRM-Sponsored PCOS Consensus Workshop Group (2004). Revised 2003 consensus on diagnostic criteria and long-term health risks related to polycystic ovary syndrome. Fertil. Steril..

[19] Kakoly N.S., Khomami M.B., Joham A.E., Cooray S.D., Misso M.L., Norman R.J., Moran L.J. (2018). Ethnicity, obesity and the prevalence of impaired glucose tolerance and type 2 diabetes in PCOS: A systematic review and meta-regression. Hum. Reprod. Update.

[20] Kahal H., Kyrou I., Uthman O.A., Brown A., Johnson S., Wall P.D., Randeva H.S. (2020). The prevalence of obstructive sleep apnoea in women with polycystic ovary syndrome: A systematic review and meta-analysis. Sleep Breath..

[21] Teede H.J., Misso M.L., Costello M.F., Dokras A., Laven J., Moran L., Norman R.J. (2018). Recommendations from the international evidence-based guideline for the assessment and management of polycystic ovary syndrome. Hum. Reprod..

[22] Dokras A., Saini S., Gibson-Helm M., Schulkin J., Cooney L., Teede H. (2017). Gaps in knowledge among physicians regarding diagnostic criteria and management of polycystic ovary syndrome. Fertil. Steril..

[23] Gibson-Helm M., Dokras A., Karro H., Piltonen T., Teede H.J. (2018). Knowledge and Practices Regarding Polycystic Ovary Syndrome among Physicians in Europe, North America, and Internationally: An Online Questionnaire-Based Study. Semin. Reprod. Med..

[24] Moin A.S.M., Sathyapalan T., Butler A.E., Atkin S.L. (2021). Vitamin D Association With Macrophage-Derived Cytokines in Polycystic Ovary Syndrome: An Enhanced Risk of COVID-19 Infection?. Front. Endocrinol..

[25] Subramanian A., Anand A., Adderley N.J., Okoth K., Toulis K.A., Gokhale K., Nirantharakumar K. (2021). Increased COVID-19 infections in women with polycystic ovary syndrome: A population-based study. Eur. J. Endocrinol..

[26] Snyder B.S. (2006). The lived experience of women diagnosed with polycystic ovary syndrome. J. Obstet. Gynecol. Neonatal Nurs..

[27] Kitzinger C., Willmott J. (2002). ‘The thief of womanhood’: Women’s experience of polycystic ovarian syndrome. Soc. Sci. Med..

[28] Hillman S.C., Bryce C., Caleyachetty R., Dale J. (2020). Women’s experiences of diagnosis and management of polycystic ovary syndrome: A mixed-methods study in general practice. Br. J. Gen. Pract..

[29] Hadjiconstantinou M., Mani H., Patel N., Levy M., Davies M., Khunti K., Stone M. (2017). Understanding and supporting women with polycystic ovary syndrome: A qualitative study in an ethnically diverse UK sample. Endocr. Connect..

[30] Crete J., Adamshick P. (2011). Managing Polycystic Ovary Syndrome: What Our Patients Are Telling Us. J. Holist. Nurs..

[31] Kite C., Atkinson L., McGregor G., Clark C.C., Brown J.E., Kyrou I., Randeva H.S. (2021). Sleep disruption and depression, stress and anxiety levels in women with polycystic ovary syndrome (PCOS) during the lockdown measures for COVID-19 in the UK. Front. Glob. Women’s Health.

[32] Pfaff K.A., Baxter P.E., Jack S.M., Ploeg J. (2014). Exploring new graduate nurse confidence in interprofessional collaboration: A mixed methods study. Int. J. Nurs. Stud..

[33] Musselwhite K., Cuff L., McGregor L., King K.M. (2007). The telephone interview is an effective method of data collection in clinical nursing research: A discussion paper. Int. J. Nurs. Stud..

[34] Braun V., Clarke V. (2006). Using thematic analysis in psychology. Qual. Res. Psychol..

[35] Groarke J.M., Berry E., Graham-Wisener L., McKenna-Plumley P.E., McGlinchey E., Armour C. (2020). Loneliness in the UK during the COVID-19 pandemic: Cross-sectional results from the COVID-19 Psychological Wellbeing Study. PLoS ONE.

[36] Williams S.N., Armitage C.J., Tampe T., Dienes K. (2020). Public perceptions and experiences of social distancing and social isolation during the COVID-19 pandemic: A UK-based focus group study. BMJ Open.

[37] Grey E., Solomon-Moore E., Lambert J., Gillison F., Townsend N., Griffin T. Lockdown lifestyles: A mixed methods study exploring the impact of COVID-19 prevention measures on diet, physical activity and mental health in the UK adult population. Proceedings of the Annual General Meeting of the UK Society of Behavioural Medicine (Virtual).

[38] Tay C.T., Teede H.J., Hill B., Loxton D., Joham A.E. (2019). Increased prevalence of eating disorders, low self-esteem, and psychological distress in women with polycystic ovary syndrome: A community-based cohort study. Fertil. Steril..

[39] Lim S., Smith C.A., Costello M.F., MacMillan F., Moran L., Ee C. (2019). Barriers and facilitators to weight management in overweight and obese women living in Australia with PCOS: A qualitative study. BMC Endocr. Disord..

[40] Institute M.G. (2021). The Future of Work after COVID-19. https://www.mckinsey.com/featured-insights/future-of-work/the-future-of-work-after-covid-19.

[41] Wood S.J., Michaelides G., Inceoglu I., Hurren E.T., Daniels K., Niven K. (2021). Homeworking, well-being and the Covid-19 pandemic: A diary study. Int. J. Environ. Res. Public Health.

[42] Damone A.L., Joham A.E., Loxton D., Earnest A., Teede H.J., Moran L.J. (2019). Depression, anxiety and perceived stress in women with and without PCOS: A community-based study. Psychol. Med..

[43] Richardson S., Hirsch J.S., Narasimhan M., Crawford J.M., McGinn T., Davidson K.W. (2020). Northwell COVID-19 Research Consortium. Presenting characteristics, comorbidities, and outcomes among 5700 patients hospitalized with COVID-19 in the New York City area. JAMA.

[44] Tomlinson J., Pinkney J., Adams L., Stenhouse E., Bendall A., Corrigan O., Letherby G. (2017). The diagnosis and lived experience of polycystic ovary syndrome: A qualitative study. J. Adv. Nurs..

[45] Tippett A. (2021). Life on pause: An. analysis of UK fertility patients’ coping mechanisms after the cancellation of fertility treatment due to COVID-19. J. Health Psychol..

[46] Grabowski D., Overgaard M., Meldgaard J., Johansen L.B., Willaing I. (2021). Disrupted Self-Management and Adaption to New Diabetes Routines: A Qualitative Study of How People with Diabetes Managed Their Illness during the COVID-19 Lockdown. Diabetology.

[47] Grannell A., le Roux C.W., McGillicuddy D. (2020). “I am terrified of something happening to me” The lived experience of people with obesity during the COVID-19 pandemic. Clin. Obes..

[48] Gillard S., Dare C., Hardy J., Nyikavarand P., Rowan O.R., Shah P., BirkeN M., Foye U., Ocloo J., Pearce E. (2012). Experiences of living with mental health problems during the COVID-19 pandemic in the UK: A coproduced, participatory qualitative interview study. Soc. Psychiatry Psychiatr. Epidemiol..

[49] Burton A., McKinlay A., Aughterson H., Fancourt D. (2020). Impact of the Covid-19 pandemic on the mental health and wellbeing of adults with mental health conditions in the UK: A qualitative interview study. medRxiv.

[50] Moreno C., Wykes T., Galderisi S., Nordentoft M., Crossley N., Jones N., Cannon M., Correll U.C., Byrne L., Carr S. (2020). How mental health care should change as a consequence of the COVID-19 pandemic. Lancet Psychiatry.

