# Supplementary material for: Uncertainty, Anxiety and Isolation: Experiencing the COVID-19 Pandemic and Lockdown as a Woman with Polycystic Ovary Syndrome (PCOS)
Source: J Pers Med. 2021 Sep 25;11(10):952. doi: 10.3390/jpm11100952 (PMC8539750; doi:10.3390/jpm11100952)
Supplement: Supplementary file 1 [file jpm-11-00952-s001.zip › jpm-1385457-supplementary.pdf]

Table S1 – Participant characteristics collected via online survey

Variable	Mean ± SD or *N* (%)
**Age** (years)	31.08 ± 5.62
**Time since diagnosis** (years)	8.69 ± 5.32
**Weight** (kg)	107.38 ± 27.03
**Body mass index** (kg/m^2)	38.90 ± 9.66
**Ethnicity**	
White	10 (83)
Asian or Asian British	2 (17)
**Marital Status**	
Married	6 (50)
Single	2 (17)
Co-habiting	2 (17)
Long-term relationship	1 (8)
Civil partnership	1 (8)
**Employment status**	
Full-time employed	4 (33)
Part-time employed	2 (17)
House person	2 (17)
Student	2 (17)
Self-employed	1 (8)
Unemployed	1 (8)
**Education level**	
Undergraduate	4 (33)
Postgraduate	3 (25)
College	3 (25)
Secondary	2 (17)
**Children**	
No	7 (58)
Yes	5 (42)
**Household income**	
≤ £39,999	6 (50)
£40,000-£79,999	4 (33)
≥ £80,000	2 (17)

COVID-19 Lockdown question	*N* (%)
**Self-isolating due to COVID-19**	
No	9 (75)
Yes	3 (25)
**Difficulty following restrictions**	
No difficulty	3 (25)
Somewhat difficult	8 (67)
Very difficult	1 (8)
**Effect of lockdown on health**	
A significant negative effect	4 (33)
A small negative effect	6 (50)
No effect	-
A small positive effect	2 (17)
A significant positive effect	-
**Effect of lockdown on physical activity levels**	
A significant reduction	6 (50)
A small reduction	2 (17)
No change	-
A small increase	3 (25)
A significant increase	1 (8)
**Effect of lockdown on social interactions**	
A significant reduction	11 (92)
A small reduction	1 (8)
No change	-
A small increase	-
A significant increase	-
**Effect of lockdown on sleep**	
A significant negative effect	3 (25)
A small negative effect	7 (58)
No effect	1 (8)
A small positive effect	1 (8)
A significant positive effect	-
**Effect of lockdown on finances**	
A significant negative effect	3 (25)
A small negative effect	2 (17)
No effect	5 (42)
A small positive effect	2 (17)
A significant positive effect	-
